# Wear and Corrosion Resistance Technology of Thin Film Materials: A New Open Special Issue in *Materials*

**DOI:** 10.3390/ma15155218

**Published:** 2022-07-28

**Authors:** Sangmin An

**Affiliations:** Department of Physics, Institute of Photonics and Information Technology, Jeonbuk National University, Jeonju 54896, Korea; san@jbnu.ac.kr; Tel.: +82-63-270-3445

“Wear and Corrosion Resistance Technology of Thin Film Materials” is a new and open Special Issue published in *Materials*, presenting research and review papers that focus on the wear and corrosion resistance of various materials, new scientific issues and their useful applications in wear and corrosion research and industrial sectors.

Over the last decade, wear and corrosion resistance technology has emerged as an exciting research field, offering perspectives on how to reduce the unit prices of products and their fabrication processes in industrial sectors and driving future research and developments (R & D).

Wear and corrosion resistance technology is crucial for industrial applications, which include several scientific issues, such as surface protection [1,2], the improvement of lubricity, and even chemical resistance [3]. Furthermore, wear and corrosion resistance technology in thin film materials can effectively reduce the effect of wear and corrosion using coatings or deposition with various materials, such as composite films [4,5], physical vapor deposition [6], plasma-assisted chemical vapor deposition coating [7], plasma-enhanced chemical vapor deposition coating [8], plasma electrolytic oxidation [9], etc.

The Special Issues, entitled “Wear and Corrosion Resistance Technology of Thin Film Materials”, collates research that explores various wear and corrosion resistance technologies (development and applications), including studies on lubricants or friction, focusing on, but not necessarily limited to, the following themes: wear and corrosion resistance technologies in thin films; various materials for film coating technologies; metal alloys or carbon-based investigations of thin films; macroscopic or microscopic (micro/nano) composite films for wear and corrosion; and the development and applications of resistance technologies for thin film materials in various environments, such as semiconductor applications (LED/OLED), aerospace/automobile engineering, energy and environmental technology, medical/pharmaceutical industry, and the paper/cellulose industry.
